# Distinct Roles of Extracellular Domains in the Epstein-Barr Virus-Encoded BILF1 Receptor for Signaling and Major Histocompatibility Complex Class I Downregulation

**DOI:** 10.1128/mBio.01707-18

**Published:** 2019-01-15

**Authors:** Suzan Fares, Katja Spiess, Emma T. B. Olesen, Jianmin Zuo, Sarah Jackson, Thomas N. Kledal, Mark R. Wills, Mette M. Rosenkilde

**Affiliations:** a Laboratory for Molecular and Translational Pharmacology, Department of Biomedical Sciences, Faculty of Health and Medical Sciences, University of Copenhagen, Copenhagen, Denmark; b Department of Clinical Biochemistry, Copenhagen University Hospital, Copenhagen, Denmark; c Institute of Immunology and Immunotherapy, College of Medical and Dental Sciences, University of Birmingham, Birmingham, United Kingdom; d Division of Infectious Diseases, Department of Medicine, University of Cambridge, Cambridge, United Kingdom; e National Veterinary Institute, Technical University of Denmark, Lyngby, Denmark; University of KwaZulu-Natal

**Keywords:** EBV-BILF1, Epstein-Barr virus, GPCR, major histocompatibility complex, signaling, tumor immunology

## Abstract

The Epstein-Barr virus (EBV) BILF1 gene encodes a constitutively active G protein-coupled receptor (GPCR) that downregulates major histocompatibility complex (MHC) class I and induces signaling-dependent tumorigenesis. Different BILF1 homologs display highly conserved extracellular loops (ECLs) including the conserved cysteine residues involved in disulfide bridges present in class A GPCRs (GPCR bridge between transmembrane helix 3 [TM-3] and ECL-2) and in chemokine receptors (CKR bridge between the N terminus and ECL-3). In order to investigate the roles of the conserved residues in the receptor functions, 25 mutations were created in the extracellular domains. Luciferase reporter assays and flow cytometry were used to investigate the G protein signaling and MHC class I downregulation in HEK293 cells. We find that the cysteine residues involved in the GPCR bridge are important for both signaling and MHC class I downregulation, whereas the cysteine residues in the N terminus and ECL-3 are dispensable for signaling but important for MHC class I downregulation. Multiple conserved residues in the extracellular regions are important for the receptor-induced MHC class I downregulation, but not for signaling, indicating distinct structural requirements for these two functions. In an engineered receptor containing a binding site for Zn^+2^ ions in a complex with an aromatic chelator (phenanthroline or bipyridine), a ligand-driven inhibition of both the receptor signaling and MHC class I downregulation was observed. Taken together, this suggests that distinct regions in EBV-BILF1 can be pharmacologically targeted to inhibit the signaling-mediated tumorigenesis and interfere with the MHC class I downregulation.

## Abstract (precis)

G protein-coupled receptors constitute the largest family of membrane proteins. As targets of >30% of the FDA-approved drugs, they are valuable for drug discovery. The receptor is composed of seven membrane-spanning helices and intracellular and extracellular domains. BILF1 is a receptor encoded by Epstein-Barr virus (EBV), which evades the host immune system by various strategies. BILF1 facilitates the virus immune evasion by downregulating MHC class I and is capable of inducing signaling-mediated tumorigenesis. BILF1 homologs from primate viruses show highly conserved extracellular domains. Here, we show that conserved residues in the extracellular domains of EBV-BILF1 are important for downregulating MHC class I and that the receptor signaling and immune evasion can be inhibited by drug-like small molecules. This suggests that BILF1 could be a target to inhibit the signaling-mediated tumorigenesis and interfere with the MHC class I downregulation, thereby facilitating virus recognition by the immune system.

## INTRODUCTION

Epstein-Barr virus (EBV) infects ∼90% of adults worldwide (1–3). The virus is classified as a gamma 1 gammaherpesvirus in genus *Lymphocryptovirus* (LCV) and forms latent infection in B cells after the establishment of a balance between host immune response and virus immune evasion strategies. The virus is associated with growth-transforming activity in human B cells resulting in different types of cancers (4–6). In order to succeed in efficient immune evasion and establish a lifelong infection, EBV genes encode a number of immunoevasins, some of which target the major histocompatibility complex (MHC) class I molecules, including BGLF5 and BNLF2a. The virus is normally kept under T cell surveillance, yet the immunoevasins compromise the elimination of the EBV-transformed cells, thereby contributing to malignancies associated with EBV infection (4, 5, 7–9). Among these EBV-encoded immunoevasins is BILF1, a viral gene encoding a G protein-coupled receptor (GPCR). Several functions have been shown for herpesvirus-encoded GPCRs (vGPCRs) including chemokine scavenging, cell migration stimulation, and intracellular signaling reprogramming (2, 10). These genes are believed to be captured through molecular piracy during coevolution of the virus with the respective host (11–13).

The EBV-encoded BILF1 receptor downregulates MHC class I and induces signaling-mediated tumorigenesis both *in vitro* and *in vivo* (14–17). It signals constitutively via Gα_i_, where it modulates cyclic AMP (cAMP) response element (CRE) activation (18, 19). This constitutive activity is shared with other virus-encoded receptors such as US28 (human cytomegalovirus [HCMV]) and ORF74 (human herpesvirus 8 [HHV8]), both of which also induce signaling-mediated tumorigenesis (10, 13, 20–22). Unlike these vGPCRs, BILF1 has a unique immunomodulatory function, where it downregulates MHC class I surface expression (15–17), which causes marked impairment of T cell recognition (16, 17). BILF1 is a late lytic EBV protein (8). Its immunosuppressive activity increases as the lytic cycle of EBV progresses and it predominantly inhibits T cell recognition at the late phase of the virus lytic cycle (8). BILF1 expression has also been detected at low levels during latency in Burkitt’s lymphoma cell lines (23). Moreover, it has been detected in EBV-transformed B cell lines (8, 23).

Sequence alignment of 21 BILF1 homologs (15 closely related primate gamma 1 gammaherpesvirus-encoded BILF1 sequences and 6 sequences from the ungulate gammaherpesviruses) revealed a high degree of conservation among the extracellular loops (ECLs), especially ECL-2 (24). Among the conserved residues were the cysteine residues believed to be involved in the GPCR and chemokine characteristic disulfide bridge (24).

GPCRs are targets of more than 30% of marketed drugs with orphan receptors representing great opportunities in the treatment of many diseases (25). Nearly 400 small molecules are currently being investigated as active therapeutics for around 100 human GPCRs (25). Metal ion site engineering in GPCRs has been used as a tool for decades to study helical connectivity and provide knowledge about distance constraints and conformational changes in GPCRs. This strategy was first implemented in 1995, when Elling et al. (26) reported the conversion of the antagonist binding site in the human tachykinin NK1 receptor to a high-affinity metal ion binding site by substituting the antagonist binding site with histidine residues. Several studies followed using metal ion site engineering to probe putative binding pockets for small molecules in GPCRs (27–34). The BILF1 receptor does not resemble any other GPCR, and therefore, current X-ray and Cryo-EM structures are not applicable for building homology models. The metal ion site engineering in BILF1 is therefore a valuable strategy to predict a putative ligand binding pocket in the receptor. BILF1 signaling activity has been linked to tumor formation, and it was therefore suggested as a potential drug target against virus-induced cancers through receptor signaling inhibition by small-molecule antagonists or inverse agonists (14). In the current study, we aimed to investigate the possibility of targeting the BILF1 receptor using small molecules in order to modulate both its signaling and immune evasion functions. We used different approaches to investigate sequence elements and predict helical connectivity of the receptor. On the basis of amino acid residue conservation, we created and characterized 25 EBV-BILF1 extracellular domain mutants in terms of MHC class I downregulation and signaling. We also engineered the receptor to contain a binding site for small ‘‘model’’ compounds, the metal ion chelators (32–34). We tested the aromatic chelators bipyridine and phenanthroline, in a complex with Zn^+2^, for their ability to modulate receptor signaling and MHC class I downregulation.

## RESULTS

**FIG 1:**
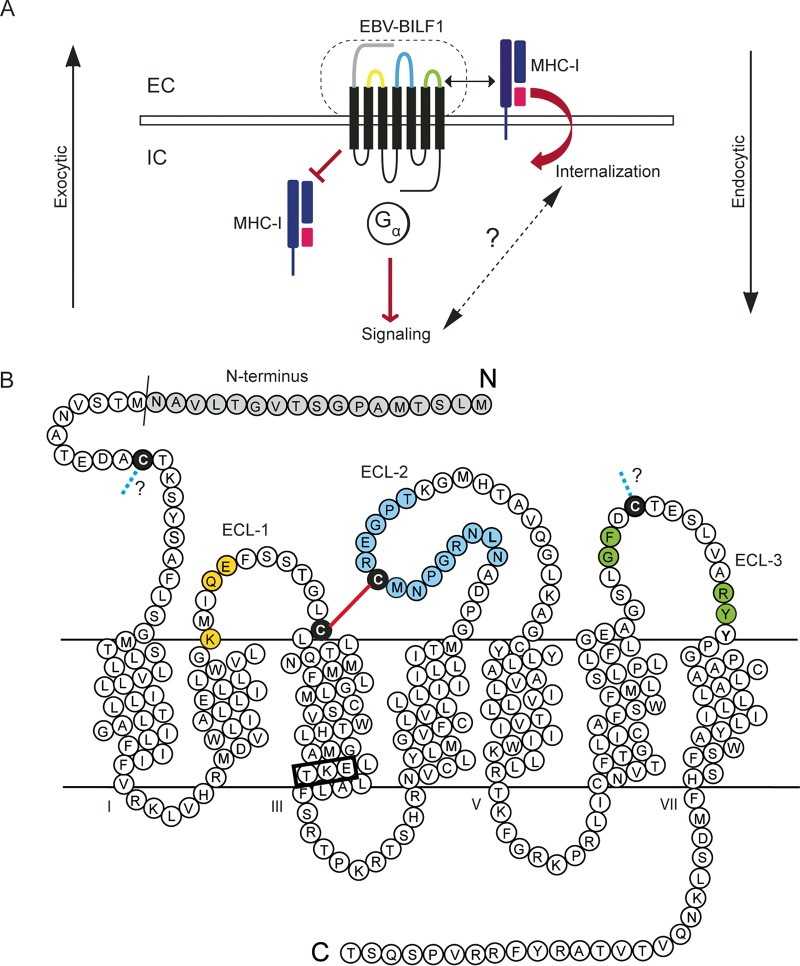
Model of EBV-BILF1 and its role in MHC class I downregulation. (A) In the endocytic pathway, EBV-BILF1 physically associates with MHC class I at the cell surface and enhances MHC class I internalization. In the exocytic pathway, EBV-BILF1 inhibits the appearance of newly synthesized MHC class I at the cell surface. EC, extracellular; IC, intracellular. (B) Serpentine diagram of EBV-BILF1, showing the conserved amino acids in yellow in ECL-1, in blue in ECL-2, and in green in ECL-3 (mutated to alanine in this study). The four conserved cysteine residues (mutated to alanine in this study) are shown in black. The putative GPCR bridge is shown in red, and the chemokine receptor bridge (CKR bridge) is shown as a blue dotted line. The N-terminal residues that were deleted in this study to create the mutant Δ17-N-term are shown in gray. The DRY-like EKT signaling motif is marked by a black box at the bottom of TM-3.

BILF1 is an immune evasion gene that modulates the immune system by downregulating MHC class I via the endocytic pathway, where MHC class I is targeted for internalization and the exocytic pathway, where it inhibits the appearance of newly synthesized MHC molecules at the cell surface (16, 17) (Fig. 1A). We have previously described a high degree of conservation among the ECLs of BILF1 receptors, especially ECL-2 (24). In order to investigate the roles of these conserved residues in the receptor-induced signaling and MHC class I downregulation, we generated 24 point mutations in the EBV-BILF1 extracellular domain and a mutant, where the first 17 residues of the receptor N terminus were deleted (Δ17-N-term) (Fig. 1B). A total of 25 EBV-BILF1 mutants were generated in the current study and investigated for surface expression, G protein-mediated signaling, and MHC class I downregulation in HEK293 cells (Fig. 1B).

### Impact of conserved cysteine residues in extracellular receptor regions of EBV-BILF1.

**FIG 2:**
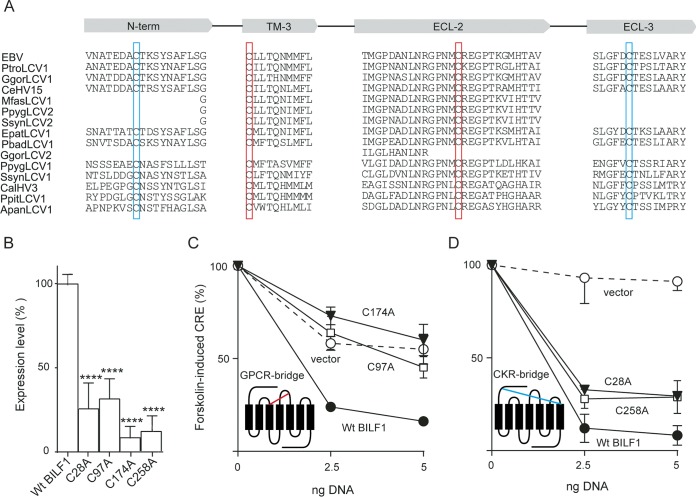
Sequence alignment of different BILF1 homologs and expression and signaling of EBV-BILF1 mutants without disulfide bridges. (A) Fifteen primate BILF1 receptors were aligned using MAFFT (Geneious 7). The sequences show four domains of BILF1, the N terminus (N-term), transmembrane 3 (TM-3), and extracellular loops 2 (ECL-2) and 3 (ECL-3). The cysteine residues involved in the formation of the GPCR bridge are shown in red boxes. The cysteine residues involved in the formation of the CKR bridge are shown in blue boxes. (B) ELISA showing the cell surface expression level of four cysteine mutants (C28A, C97A, C174A, and C258A). HEK293 cells were transiently transfected with 15 ng of N-terminally HA-tagged receptor in the pCMV-HA vector. One day after transfection, the cells were fixed in 4% paraformaldehyde and incubated with the antibodies. The optical density (OD) was measured at 450 nm, and the data were normalized to empty vector (0%) and wild-type (Wt) BILF1 (100%) and analyzed by the paired Student *t* test. Values that are significantly different (*P* < 0.0001) from the value from the Wt BILF1 are indicated by four asterisks. (C and D) Gα_i_ signaling activity in HEK293 cells cotransfected with receptor/empty vector DNA, pFR-Luc transactivator plasmid, or pFA2-CRE transreporter plasmid. Twenty-four hours after transfection, the cells were induced with forskolin (15 µM) for 5 h to activate CRE (via adenylate cyclase activation). (C) Cells transfected with different concentrations of C97A (open squares), C174A (closed triangles), empty pCMV-HA vector (open circles), or wt BILF1 (closed circles) plasmid DNA. The GPCR bridge between the two cysteine residues C97 (TM-3) and C174 (ECL-2) is shown in red. (D) Cells transiently transfected with different concentrations of C258A (open squares), C28A (closed triangles), empty pCMV-HA vector (open circles) or wt BILF1 (closed circles) plasmid DNA. The CKR bridge between the two cysteine residues C28 (N-term) and C258 (ECL-3) is shown in blue. The figure is from 3 independent experiments normalized to the values in the absence of forskolin (0%) and maximum activity (100%). Error bars represent standard errors of the means (SEM). The error bars for wt BILF1 in panel C were smaller than the symbols and are therefore not visible.

The GPCR characteristic disulfide bridge (GPCR bridge) between the conserved cysteine residues of TM-3 and ECL-2 is a common structural feature among GPCRs (35). EBV-BILF1 displays two cysteine residues in position 97 (C97) at the top of transmembrane helix 3 (TM-3) and in position 174 (C174) in ECL-2 (Fig. 2A, red; Fig. 1B, black). These residues are conserved among BILF1 receptors (24) (Fig. 2A, red). We investigated the impact of these two residues on the receptor functions. To this end, both residues were mutated to alanine (C97A and C174A), and their surface expression and signaling were investigated. Both mutants showed reduced surface expression measured by surface ELISA against the N-terminally attached HA tag with expression of ∼30% (C97A) and 10% (C174A) of that of wild-type (wt) EBV-BILF1 (Fig. 2B). ELISA and flow cytometry are commonly used techniques to measure the surface expression, internalization, and recycling of GPCRs (24, 36–39).

The Gα_i_ signaling activity was investigated through their ability to modulate the forskolin-induced CRE. Forskolin activates adenylate cyclase (AC), which induces cAMP formation that will result in the downstream activation of CRE, which is inhibited by Gα_i_-coupled receptors (EBV-BILF1 in this case). The C97A and C174A mutants could not inhibit the forskolin-induced CRE compared to wt EBV-BILF1, indicating an abolished Gα_i_ signaling activity (Fig. 2C). This phenotype was confirmed by cotransfection with Gα_qi4myr_ in CRE-luciferase assay (see Fig. S1A in the supplemental material). Gα_qi4myr_ provides Gα_i_ coupling/Gα_q_ signaling system, activating CRE through phospholipase C (PLC). In other words, the Gα_i_-coupled BILF1 receptor recognizes this chimeric protein as a Gα_i_, but it functions as Gα_q_ subunit. Both mutants did not activate CRE in Gα_qi4myr_-cotransfected HEK293 cells (Fig. S1A).

10.1128/mBio.01707-18.1 FIG S1 Signaling of EBV-BILF1 mutants without disulfide bridges in cells cotransfected with Gα_qi4myr_ (gives Gα_q_ readout activating CRE). (A and B) Gα_i_ signaling shown by the induction of CRE in cells cotransfected with BILF1 and Gα_qi4myr_. HEK293 cells were cotransfected with receptor/empty vector DNA, pFR-Luc transactivator plasmid, and pFA2-CRE transreporter plasmid and Gα_qi4myr_. (A) CRE activity measured in cells transiently transfected with different concentrations of C97A or C174A (open squares), empty vector (stippled), or wt BILF1 (closed circles) plasmid DNA. (B) CRE activity measured in cells transfected with different concentrations of C28A or C258A (open squares), empty vector (stippled), or wt EBV-BILF1 (closed circles) plasmid DNA. Error bars represent standard errors of the means (SEM) from three independent experiments normalized to the maximum activity. Download FIG S1, PDF file, 0.8 MB. Copyright © 2019 Fares et al. 2019 Fares et al. This content is distributed under the terms of the Creative Commons Attribution 4.0 International license.

EBV-BILF1 displays two additional cysteine residues in the N terminus (C28) and ECL-3 (C258) (Fig. 2A, blue; Fig. 1B, black). These residues are conserved among BILF1 receptors (24) (Fig. 2A, blue). We investigated the roles of these two residues in receptor signaling and surface expression. The two residues were mutated to alanine. The mutants showed reduced surface expression of ∼25% (C28A) and 12% (C258A) of that of wt EBV-BILF1 (Fig. 2B). However, both mutants retained a wt-like Gα_i_ signaling activity (Fig. 2D), which was confirmed by the induction of CRE in Gα_qi4myr_-cotransfected HEK293 cells (Fig. S1B). This suggests that low receptor expression *per se* does not result in low signaling and that these two cysteine residues are not important for the receptor signaling.

### Roles of conserved residues in extracellular BILF1 regions.

**FIG 3:**
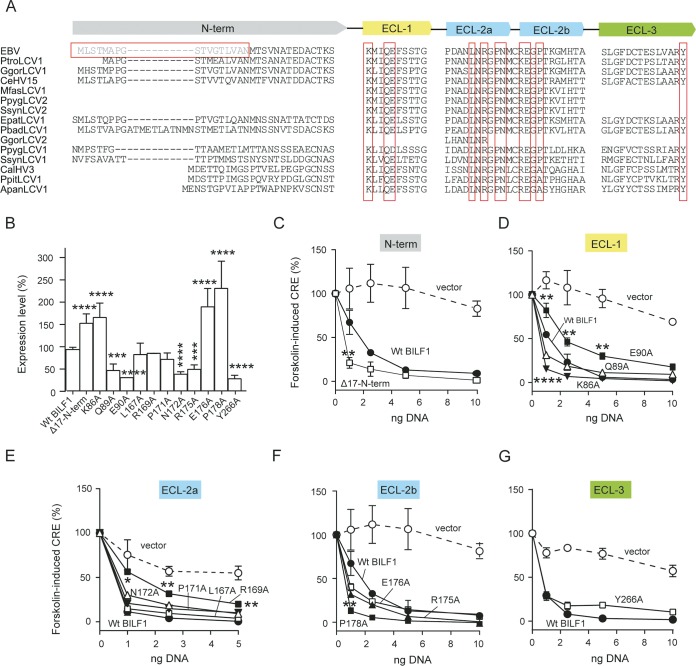
Sequence alignment of different BILF1 homologs and expression and signaling of different EBV-BILF1 extracellular domain mutants. (A) The sequences were created as described in the legend to Fig. 2A. The sequences show the different regions of the BILF1 extracellular domain, the N terminus (N-term; gray) and extracellular loops 1 (ECL-1; yellow), 2 (ECL-2; blue), and 3 (ECL-3; green). Conserved amino acids (mutated to alanine in the current study) are shown in red boxes. The N-terminus deletion mutant (Δ17-N-term) is shown in light gray in a red box. (B) ELISA showing the cell surface expression level as in Fig. 2. (C to G) Gα_i_ signaling as in Fig. 2. HEK293 cells were transfected with a concentration range of 1 to 10 ng of N-terminally HA-tagged receptor in the pCMV-HA vector. Error bars represent standard errors of the means (SEM) for 3 or 4 independent experiments normalized to the values in the absence of forskolin (0%) and maximum activity (100%). Values that were significantly different by the paired Student *t* test are indicated by asterisks as follows: *, P < 0.05; **, P < 0.01; ***, P < 0.001; ****, P < 0.0001. The errors bars were smaller than the symbols in some of the figures and are therefore not visible.

In order to investigate the roles of other BILF1 extracellular domain conserved residues (Fig. 3A) in receptor signaling and surface expression, we created 20 point mutations (Fig. 1B) located in ECL-1 (3 mutations [Fig. 1B, yellow]), ECL-2 (13 mutations [Fig. 1B, blue]), and ECL-3 (4 mutations [Fig. 1B, green]), and an N-terminal deletion (Fig. 1B, gray). These residues are marked with red boxes in Fig. 3A. Alanine was introduced in those conserved positions in the ECLs, and the first 17 residues of the EBV-BILF1 N terminus (Δ17-N-term) were deleted (Fig. 1B and 3A). The surface expression measured by ELISA and signaling of all these mutants are shown in Fig. 3 and Fig. S2. The mutants showed differential expression levels (Fig. 3B). In ECL-2a, L167A, R169A, and P171A mutants showed a wt-like surface expression level, whereas two mutants, mutants with N172A and R175A located in a closer proximity to the conserved cysteine (C174), showed lower expression than wt EBV-BILF1 did (Fig. 3B). The mutants with Q89A and E90A in ECL-1 and Y266A in ECL-3 also showed reduced expression. In contrast, the deletion of the N terminus residues (Δ17-N-term), removing the positive charge (K86A) in ECL-1 and substituting E176 and P178 in ECL-2b by alanine resulted in higher surface expression than that for wt EBV-BILF1 (Fig. 3B). The Gα_i_ signaling activity for all the mutants was retained (Fig. 3C to G). Three mutants, Δ17-N-term, K86A, and P178A mutants, showed even higher activity compared to wt EBV-BILF1 at low DNA concentrations (Fig. 3C, D, and F), whereas two mutants, E90A and R169A mutants, showed reduced signaling activity compared to wt EBV-BILF1 (Fig. 3D and E). Figure 3 shows the surface expression and signaling of 12 mutants; the rest (9 mutants) are shown in Fig. S2. The Gα_i_ signaling activity of the mutants displayed in Fig. 3 and Fig. S2 was confirmed by cotransfection of Gα_qi4myr_ in CRE-luciferase assay (Fig. S3).

10.1128/mBio.01707-18.2 FIG S2 Signaling and expression of different EBV-BILF1 mutants. (A) ELISA showing the cell surface expression level of different BILF1 mutants. (B to F) Gα_i_ signaling shown by the inhibition of forskolin-induced CRE. The experiments were performed as described in the legend to Fig. 2. The figure is from three independent experiments normalized to the values in the absence of forskolin (0%) and maximum activity (100%). Error bars represent SEM. The error bars were smaller than the symbols in some of the panels and therefore were not visible. Download FIG S2, PDF file, 0.8 MB. Copyright © 2019 Fares et al. 2019 Fares et al. This content is distributed under the terms of the Creative Commons Attribution 4.0 International license.

10.1128/mBio.01707-18.3 FIG S3 Gα_i_ signaling of different EBV-BILF1 mutants in cells cotransfected with Gα_qi4myr_. The experiments were performed as described in the legend to Fig. S1. Wt BILF1 is represented by closed symbols, the mutants are shown by open symbols, and vector is shown by stippled lines. Error bars represent SEM for three independent experiments normalized to the maximum activity. Download FIG S3, PDF file, 1.0 MB. Copyright © 2019 Fares et al. 2019 Fares et al. This content is distributed under the terms of the Creative Commons Attribution 4.0 International license.

### Impaired MHC class I downregulation upon removal of the conserved residues in extracellular BILF1 regions.

**FIG 4:**
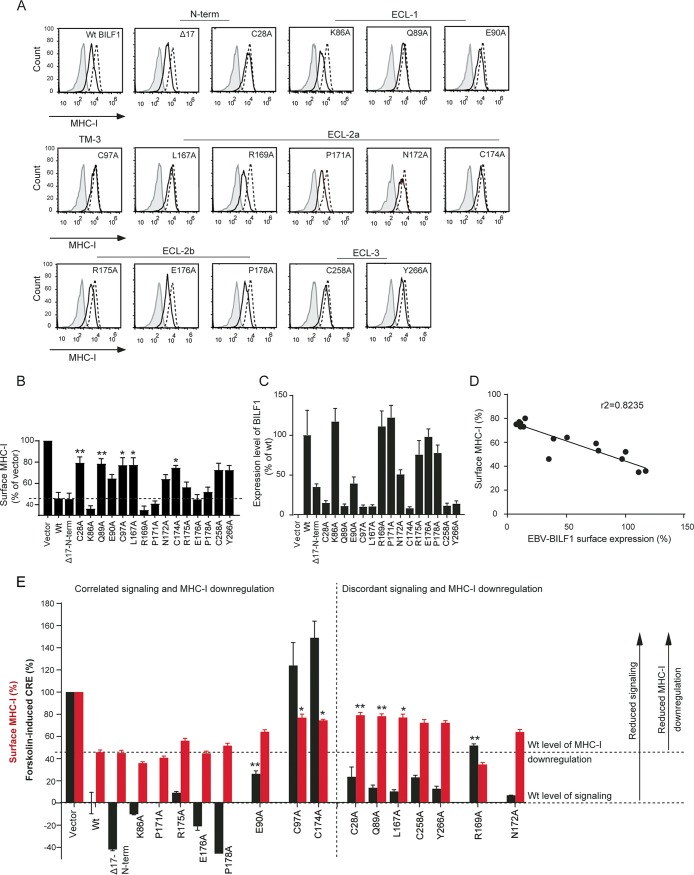
Flow cytometry showing MHC class I downregulation by different EBV-BILF1 mutants and its correlation with signaling and surface expression. (A) Histograms for the MHC class I downregulation by EBV-BILF1 mutants. HEK293T cells were transiently transfected with 1 µg of pcDNA3-HABILF1-IRES-nlsGFP (solid black line) or empty pcDNA-IRES-nlsGFP vector (broken black line). Forty-eight hours posttransfection, the cells were harvested and analyzed by flow cytometry. The isotype control staining is shown in gray. The cells were gated at high GFP and high HA expression. (B) Bar charts showing the MHC class I downregulation by the EBV-BILF1 mutants shown in Fig. 4A, normalized to the mean anti-MHC class I fluorescence index of the empty vector. (C) Surface expression level measured by mean anti-HA fluorescence index and normalized to the vector (0%) and wild-type (Wt) BILF1 (100%). (D) Correlation between the EBV-BILF1 receptor surface expression and MHC class I surface level. (E) Signaling and MHC class I downregulation activity depicted in pairs. The black bars show the signaling activity of 2.5 ng DNA concentration normalized to the vector (100%) and wt BILF1 (0%), The red bars show the MHC class I downregulation normalized as in panel B. Error bars represent SEM of ≥3 independent experiments analyzed by the paired Student *t* test (*, P < 0.05; **, P < 0.01).

We then proceeded with the investigation of the MHC class I downregulation function of the mutants displayed in Fig. 2 (4 mutants) and Fig. 3 (12 mutants) using flow cytometry (Fig. 4A). These mutants were selected based on their degree of conservation, position in the receptor, and their signaling and expression patterns. A representative experiment for MHC class I downregulation is shown in Fig. 4A, while Fig. 4B shows the values quantified from five independent experiments. Consistent with what has previously been shown (16, 17), wt EBV-BILF1 induced ∼50% reduction in the cell surface level of MHC class I relative to cells transfected with empty vector (Fig. 4A and B). The cysteine mutants C97A, C174A, C28A, and C258A showed impaired MHC class I downregulation, where they reduced MHC class I surface level by ∼20% (Fig. 4A and B). The Δ17-N-term, P171A, R175A, E176A, and P178A mutants induced a wt-like level of MHC class I downregulation (Fig. 4A and B). The K86A and R169A mutants induced a slightly, but reproducibly higher MHC class I downregulation than wt EBV-BILF1, where they reduced MHC class I surface level by ∼60% (Fig. 4A and B). The mutants E90A and N172A resulted in ∼40% reduction of MHC class I surface level (Fig. 4A and B). The Q89A, L167A, and Y266A mutants showed impaired MHC class I downregulation and reduced MHC class I surface level by only ∼20% (Fig. 4A and B). These results indicate that various conserved residues at the receptor extracellular domain are important for the EBV-BILF1-induced surface MHC class I downregulation.

### MHC class I downregulation is correlated with EBV-BILF1 surface expression, but not signaling activity.

EBV-BILF1 has been suggested to physically associate with the MHC class I molecules at the cell surface with further internalization of the molecules (17). We used flow cytometry to measure the surface expression of the receptor mutants (Fig. 4C) along with the MHC class I surface level in HEK293 cells expressing different BILF1 mutants. Both the surface expression and MHC class I downregulation of the mutants were correlated with *r*^2^ > 0.5 (Fig. 4D). The only mutant whose MHC class I downregulation was not correlated with its surface expression was the Δ17-N-term mutant (Fig. 4B to D). This mutant showed a wt-like level of MHC class I downregulation (Fig. 4A and B), but its surface expression measured by flow cytometry (Fig. 4C) was reduced relative to the wt. This could be because of improper folding of the N-terminally attached HA tag of the receptor mutant in the pCDNA3-IRES-nlsGFP and not pCMV-HA expression vector (see surface ELISA [Fig. 3B]). These results suggest that the receptor surface expression is important for the MHC class I downregulation function and implies a physical association between the receptor and MHC class I molecules at the cell surface as previously suggested (17).

BILF1 signaling activity, which has been shown to be related to the receptor-induced tumorigenesis (14), has not been consistently correlated with the receptor-induced MHC class I downregulation (16, 17). A possible link between these two functions was investigated in the current study. The Δ17-N-term, K86A, E90A, C97A, P171A, C174A, R175A, E176A, and P178A mutants showed correlated signaling and MHC class I downregulation functions (Fig. 4E), where Δ17-N-term, K86A, P171A, R175A, E176A, and P178A mutants retained a wt-like signaling and MHC class I downregulation functions (Fig. 4E). The E90A mutant, whose signaling activity was reduced also showed reduced MHC class I downregulation relative to the wt. Both the signaling activity and MHC class I downregulation functions of the C97A and C174A mutants were impaired (Fig. 4E). However, the C28A, Q89A, L167A, R169A, N172A, C258A, and Y266A mutants showed discordant signaling and MHC class I downregulation functions, where C28A, Q89A, L167A, C258A, and Y266A mutants that retained a wt-like signaling showed impaired MHC class I downregulation (Fig. 4E). In contrast, the R169A mutant, which showed reduced signaling activity (Fig. 3E and Fig. 4E), showed increased downregulation of MHC class I surface level by 60% compared to 50% for wt EBV-BILF1 (Fig. 4A and B). On the other hand, the N172A mutant retained a wt-like signaling and showed reduced MHC class I downregulation (Fig. 4E) relative to the wt. These results indicate that EBV-BILF1-induced MHC class I downregulation is not necessarily dependent on the signaling function of the receptor.

### EBV-BILF1-mediated functions can be inhibited by aromatic metal ion chelators.

**FIG 5:**
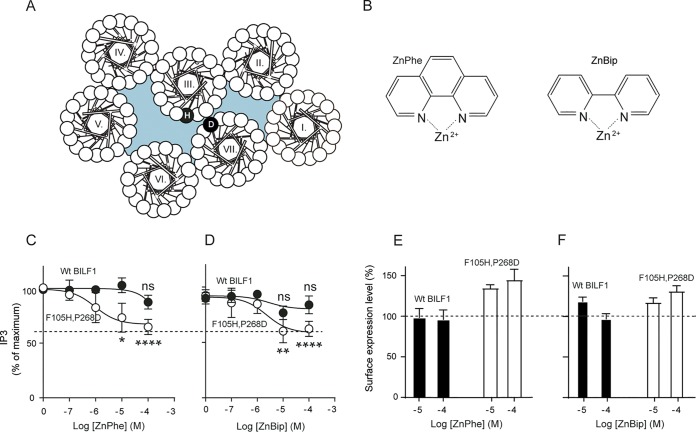
Effects of Zn^+2^ chelators on the signaling activity and expression of wt EBV-BILF1 and the F105H P268D double mutant EBV-BILF1. (A) Wheel diagram for EBV-BILF1 showing the F105H P268D double mutant. (B) Structure of phenanthroline (Phe) or bibyridine (Bip) in a complex with Zn^+2^ (ZnPhe and ZnBip). (C and D) IP3 accumulation in HEK293 cells cotransfected with wt pcDNA3-HABILF1-IRES-nlsGFP (closed circles) or the F105H P268D double mutant EBV-BILF1 (open circles) and Gα_qi4myr_, in the presence of ZnPhe (C) or ZnBip (D). The data in panels C and D were normalized to zero and maximum IP3 accumulation after background subtraction. (E and F) Surface expression level measured by mean anti-HA fluorescence index of wt BILF1 (black bars) or F105H P268D mutant (open bars) in the presence of ZnPhe (E) or ZnBip (F). The data in panels E and F were normalized to the values in the absence of the metal chelator. Error bars represent SEM from three independent experiments analyzed by the unpaired Student *t* test. Statistical significance is indicated as follows: *, P < 0.05; **, P < 0.01; ****, P < 0.0001; ns, not significant.

No ligands have been identified for EBV-BILF1, whose signaling activity has been linked to tumor formation (14). In order to investigate the possibility of the manipulation of the (i) receptor signaling activity or (ii) MHC class I downregulation function by small molecules, we created an artificial binding site in the receptor by introducing histidine (H) in position 105 (3.33 using the Ballesteros-Weinstein nomenclature [40]) and aspartic acid (D) in position 268 (7.50) to generate the F105H P268D double mutant EBV-BILF1 (Fig. 5A). The effects of the metal ion chelators (phenanthroline or bipyridine in a complex with Zn^+2^, referred to here as ZnPhe and ZnBip, respectively [Fig. 5B]) on the Gα_i_ signaling activity of the mutant compared to wt EBV-BILF1 were investigated in an inositol phosphate (IP3) accumulation assay in HEK293 cells cotransfected with Gα_qi4myr_. In wt EBV-BILF1, none of the ligands altered the basal activity of the receptor (Fig. 5C and D). In the F105H P268D double mutant, ZnPhe and ZnBip inhibited the basal activity with an EC_50_ of 1 and 2 µM, respectively, indicating inverse agonism activity of the metal chelators (Fig. 5C and D). Both ligands inhibited the receptor activity to a submaximal level (∼30%), and the level of activity depended on complex formation between Zn^+2^ and the aromatic chelators as shown in Fig. S4, where no difference was observed between the signaling activity of the wt and F105H P268D double mutant EBV-BILF1 by ZnCl_2_. Moreover, both ZnPhe and ZnBip increased the F105H P268D double mutant EBV-BILF1 surface expression by ∼40% and 30%, respectively, at the highest concentration of the metal chelator (Fig. 5E and F), whereas the effect on wt EBV-BILF1 expression was modest for ZnBip (∼15% at 10 µM) and absent for ZnPhe (Fig. 5E and F).

10.1128/mBio.01707-18.4 FIG S4 Effect of ZnCl_2_ on the signaling activity of wt EBV-BILF1 and the F105H P268D double mutant EBV-BILF1. The figure shows IP3 accumulation in HEK293 cells cotransfected with wt BILF1 (closed circles) or F105H P268D double mutant (open squares) and Gα_qi4myr_ in the presence of ZnCl_2_. Error bars represent SEM from three independent experiments normalized to the maximum IP3 accumulation. Download FIG S4, PDF file, 0.8 MB. Copyright © 2019 Fares et al. 2019 Fares et al. This content is distributed under the terms of the Creative Commons Attribution 4.0 International license.

**FIG 6:**
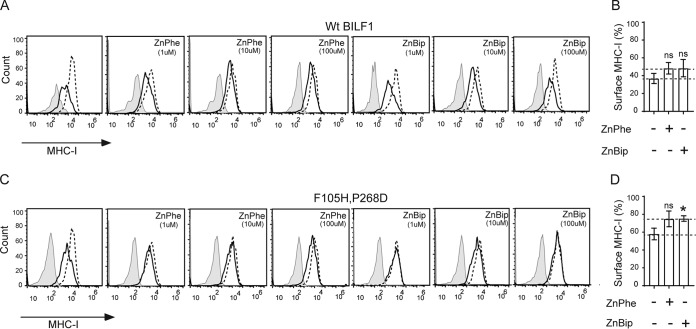
Flow cytometry showing the effects of Zn^+2^ chelators on the MHC class I downregulation of wt EBV-BILF1 and the F105H P268D double mutant EBV-BILF1. (A to D) MHC class I downregulation by wt BILF1 and F105H P268D double mutant. (A and C) Histograms for the MHC class I downregulation in cells transected with wt BILF1 (A) and F105H P268D double mutant. The transfected cells were treated with ZnPhe and ZnBip. HEK293T cells were transiently transfected with 1 µg of pcDNA3-HABILF1-IRES-nlsGFP (solid black line), empty pcDNA-IRES-nlsGFP vector (broken black line). At 24 h posttransfection, the metal chelators were added. At 48 h posttransfection, the cells were harvested, stained, and analyzed by flow cytometry. The isotype control staining is shown in gray. The cells were gated at high GFP and high HA expression. (B and D) Bar charts showing the MHC class I downregulation in cells transfected with wt BILF1 (B) and F105H P268D double mutant (D and treated with 10 µM ZnPhe or 100 µM ZnBip. Error bars represent SEM from five independent experiments normalized to vector with and without the chelators and analyzed by the unpaired Student *t* test (ns, not significant; *, P < 0.05).

The effects of the chelators were further extended to the MHC class I downregulation function of the F105H P268D double mutant EBV-BILF1 compared to wt EBV-BILF1 (Fig. 6), where different concentrations (1, 10, and 100 µM) of the ligands were assessed. Both ligands slightly inhibited the MHC class I downregulation function of wt BILF1 (Fig. 6A and B) with ∼10% higher (from 40% to 50%) MHC class I surface level relative to that in the absence of ligands (Fig. 6B). The ligands inhibited the MHC class I downregulation function of the F105H P268D double mutant EBV-BILF1 (Fig. 6C and D) with ∼15% higher (from 60% to 75%) MHC class I surface expression relative to the level in the absence of ligands (Fig. 6D).

## DISCUSSION

GPCRs possess highly diverse extracellular domains responsible for binding diverse ligands and more conserved transmembrane and intracellular domains responsible for signal transduction and binding/activating G protein and arrestins (41, 42). Interestingly, BILF1 receptors of different gammaherpesviruses display a high degree of conservation among the ECLs, a level of conservation that exceeds that of the TM region (24). In this study, we aimed to investigate the roles of the conserved residues in the EBV-BILF1 extracellular domain in relation to both receptor-mediated signaling and MHC class I downregulation, given the high degree of conservation and general involvement of the extracellular receptor domains in ligand binding. We also investigated the susceptibility of EBV-BILF1 signaling and MHC class I downregulation functions to inhibition by small-molecule ligands. Doing this we are providing the first evidence that EBV-BILF1 is a promising drug target for interference with BILF1/MHC class I interaction facilitating antigen presentation of MHC class I/peptide targets to T cells, which would facilitate virus recognition.

### Disulfide bridges, constitutive signaling, and MHC class I downregulation.

Disulfide bridges in the GPCR extracellular domain are important for maintained receptor structure and function (33, 43, 44). The disulfide bridge between TM-3 and ECL-2 is a common structural feature among class A GPCRs and present in about 90% of the receptors (44, 45). Several studies have shown that a disruption of this bridge results in impaired ligand binding and reduced ligand-dependent signaling (33, 43, 44, 46, 47). In addition, disrupting this bridge disables 90% of the receptors from reaching the cell surface (33, 48). We obtained similar results, where mutating the conserved cysteine residues in TM-3 and ECL-2 (possibly involved in GPCR bridge) resulted in reduced cell surface expression and impaired receptor signaling. We also find that these residues are essential for EBV-BILF1-induced MHC class I downregulation. These observations imply that EBV-BILF1 shares this structural feature with the rest of class A GPCRs and that this bridge is important for the structure and function of the receptor.

The extra disulfide bridge displayed by chemokine receptors (CKR bridge) provides extra receptor constraints and regulates receptor function (33, 43, 49). Here, we show that the cysteine residues possibly involved in a CKR-like bridge formation are dispensable for EBV-BILF1-mediated Gα_i_ signaling, whereas both residues are important for receptor-induced MHC class I downregulation. In addition, we found that both residues were important for maintained receptor cell surface expression. These results imply that EBV-BILF1 displays the CKR bridge and that this bridge stabilizes the receptor structure important for MHC class I downregulation and surface expression, but not signaling.

### Importance of the extracellular domain in EBV-BILF1-mediated functions.

In 2011, a study by Zuo et al. (16) identified important domains involved in BILF1-induced MHC class I downregulation; they showed that the DRY-like EKT signaling motif at the bottom of TM-3 is important for enhanced endocytosis, while the deletion of the receptor C-terminal domain resulted in impaired lysosomal degradation of MHC class I molecules. The high degree of conservation among BILF1 extracellular domains (24) makes it compelling to presume a preserved function, which could be maintaining (i) the high basal receptor activity, (ii) ligand binding, and/or (iii) interaction with MHC class I molecules. We therefore created different EBV-BILF1 extracellular domain mutants and investigated their ability to mediate signaling activity and reduce the surface level of MHC class I. Most of the mutants retained a wt-like signaling activity, which implies that this function is dependent on the intracellular region of the receptor, as previously reported for BILF1 (50) and other GPCRs (41, 42). In contrast, removing the conserved residues in the receptor extracellular domain resulted in an impaired MHC class I downregulation function of EBV-BILF1. The reduced surface expression of these mutants (Fig. 5C) indicates that the conserved residues at the extracellular domain maintain a receptor structure important for the interaction with MHC class I at the cell surface.

Our results suggest that EBV-BILF1 ECLs are directly or indirectly involved in the interaction with MHC class I molecules. Intriguingly, in contrast to the impact of Cys28 (in the N terminus) on the receptor immune modulation, deleting the first 17 residues of the EBV-BILF1 N terminus did not affect receptor signaling or MHC class I downregulation.

### Correlation between BILF1-induced signaling and MHC class I downregulation.

The link between BILF1 signaling and MHC class I downregulation function has not been consistently described (16, 17). It has been shown that the signaling-deficient K122A BILF1 mutant retained the ability to downregulate the MHC class I surface expression level (17). This mutant, which has disrupted EKT (DRY-like) signaling motif and showed impaired NF-κB signaling properties, could reduce MHC class I surface levels to an extent similar to that of the wt receptor (17). It was therefore suggested that these functions are not critically related. Afterwards, it was shown that the K122A mutant could not mediate MHC class I internalization, and it was therefore suggested that the EKT motif is necessary for MHC class I-enhanced internalization through a mechanism that is probably independent of the receptor signaling activity (16). This goes in line with our findings, where we show that MHC class I downregulation and signaling activities are not necessarily dependent. Combined with previous studies (16, 17), our observations imply that the molecular mechanisms of BILF1 function(s) are complex and that further investigation of the relationship between BILF1 signaling and MHC class I downregulation properties is warranted.

### EBV-BILF1 as a drug target.

Metal ion site engineering has traditionally been used to predict the helical connectivity of GPCRs. This has been first described for the tachykinin NK1 receptor by the introduction of histidine in the non-peptide-binding site (26) and later for others (27–34). The same strategy has been implemented to describe helical connectivity of the tumorigenic virus-encoded ORF74-HHV8 receptor (31). Similarly, an engineered site for metal ion chelator complexes was created in the CXCR3 receptor by introducing a His residue at the corresponding position for the adrenergic receptor ligand binding pocket (30).

The fact that EBV-BILF1 does not resemble any of the aforementioned receptors makes the receptor probe for such compounds challenging. Nevertheless, we successfully created an artificial binding site in EBV-BILF1 through double mutant engineering between TM-3 and TM-7. Our results raise the enticing prospect of exploiting the receptor extracellular domains (including the outer transmembrane domain) to inhibit receptor signaling and interfere with receptor/MHC class I interaction.

## MATERIALS AND METHODS

### Cell lines and culture and transfection conditions.

Dulbecco’s modified Eagle’s medium (DMEM) purchased from Invitrogen, Germany, containing 10% fetal bovine serum (FBS) and 1% penicillin-streptomycin (Pen-Strep), was used to grow HEK293 cells at 37°C and 10% CO_2_. Transient transfection of HEK293 cells was performed with Lipofectamine 2000 (Invitrogen) according to the manufacturer’s instructions and with CaPO_4_ (51).

### Vector constructs and receptor expression.

EBV-BILF1 was cloned into the pCMV-HA vector (Clontech). The expression plasmid pCDNA3-HABILF1-GFP and control empty vector pCDNA3-IRES-nlsGFP have been described previously (16, 17). The EBV-BILF1 mutants were generated by QuikChange PCR using high-fidelity Pfu DNA polymerase. The constructs were verified by restriction digestion and sequence analysis.

### Antibodies.

For enzyme-linked immunosorbent assay (ELISA) experiments, anti-hemagglutinin monoclonal antibodies (anti-HA MAbs) (anti-H11, clone 16B12, mouse; HISS Diagnostics, Germany) and goat anti-mouse horseradish peroxidase-conjugated antibody purchased from Dianova, Denmark, were used. For flow cytometry, phycoerythrin (PE)-conjugated MAb, clone W6/32, (BioLegend, UK) and Alexa Fluor 647-conjugated anti-HA (Cell Signaling, UK) were used. The antibodies were diluted according to the manufacturer’s instructions.

### Recombinant G protein plasmid (Gα_qi4myr_).

Gα_qi4myr_ or Gα_Δ6qi4myr_ is a chimeric Gα_q_ subunit in which 6 amino acid residues at the N terminus were deleted, a myristoylation motif was created at the N terminus, and the Gα_i_ 4 C-terminal residues replaced the corresponding residues in the Gα_q_ subunit (52). The receptor recognizes Gα_qi4myr_ as Gα_i_ and gives Gα_q_ readout (52).

### Metal ion chelators.

The aromatic chelators, bipyridine (Bip) or phenanthroline (Phe) were made in a complex with Zn^+2^. ZnCl_2_ was mixed with either 2,2′-bipyridine or 1,10-phenanthroline (Sigma-Aldrich, USA) in the 1:2 ratio as previously described (32, 33).

### ELISA to measure cell surface expression.

The receptor cell surface expression was confirmed by ELISA conducted as described previously (24). Briefly, HEK293 cells were seeded in 96-well plates at a density of 2 × 10^5^ cells/well 24 h prior to transient transfection with N-terminal HA-tagged receptor cloned in the pCMV vector (15 ng). One day after the cells were seeded, the cells were fixed in 4% paraformaldehyde, blocked in PBS containing bovine serum albumin (BSA) at room temperature (RT). The cells were then incubated with the primary antibody for 1 h at RT, while shaking. This was followed by a washing step and incubation with the secondary antibody for 1 h at RT, while shaking. The activity was detected by 3,3′-5,5′-tetramethyl benzidine substrate (Kem-En-Tec, Denmark) and the reaction was stopped with H_2_SO_4_. The optical density (OD) was measured at 450 nm.

### cAMP response element (CRE) reporter assay.

HEK293 cells were transiently transfected 24 h after the cells were seeded at 2 × 10^5^ cells/well, with increasing amounts of the receptor/empty vector DNA, both with 25 ng/well of pFR-Luc transactivator plasmid and 6 ng/well of pFA2-CRE transreporter plasmid (Stratagene, USA) and 30 ng/well Gα_qi4myr_ plasmid DNA (kindly provided by Evi Kostenis, University of Bonn). A concentration range of 0.5 ng to 10 ng of the receptor and empty expression vector DNA was used. For forskolin stimulation, no Gα_qi4myr_ was added. The cells were treated with 15 µM forskolin (Sigma, USA) at 24 h posttransfection, and the luciferase activity was measured at 5 h after forskolin addition.

### Inositol trisphosphate (IP3) accumulation.

The assay was performed as described previously (24, 51). Briefly, HEK293 cells were transfected with 5 μg of BILF1 or empty vector DNA and 5 μg of Gα_qi4myr_. One day after transfection, the cells were seeded in a 96-well plate at 35,000 cells/well in the presence of 4 μCi of *myo*-[^3^H]inositol. One day after the cells were seeded, the cells were incubated with different concentrations of metal ion chelators in 0.1 ml Hanks’ balanced salt solution (Invitrogen, UK) supplemented with 10 mM LiCl at 37°C for 90 min. The cells were then treated as described previously (24) and measured in a top counter. Briefly, the cells were incubated in formic acid (10 mM) on ice for 30 min. The extract was transferred to a 96-well plate and incubated with YSi poly-d-lysine-coated beads (PerkinElmer, USA) diluted 1:8 while shaking at maximum speed. The radiation was measured in a Top-Counter.

### Flow cytometry to analyze surface MHC class I molecules and receptors.

PE-labeled W6/32 antibodies or a PE-labeled isotype control MAb were used to determine the levels of total MHC class I at HEK293T cell surface. The cells were seeded in 24-well plate 24 h prior to transient transfection with 1 µg/well of the N-terminal HA-tagged BILF1 inserted in the pCDNA3-IRES-nlsGFP expression vector. Forty-eight hours after transfection, the cells were harvested and stained with anti-MHC class I and anti-HA antibody (detecting the surface level of BILF1). Zombie Red dye (BioLegend, UK) was used to stain for the live/dead (LD) cells. When the metal ion chelators were used, they were added at different concentrations at 24 h posttransfection. The samples were analyzed on a BD Accuri C6 instrument. The data were analyzed using FlowJo (TreeStar) and BD Accuri C6 software.

### Data analysis and statistics.

The data were analyzed with GraphPad Prism, FlowJo (TreeStar), and BD Accuri C6 software and expressed as means ± standard errors of the means (SEM). Statistical analysis was performed in GraphPad Prism. Specific tests are noted in the figure legends. *P* values of < 0.05 were considered statistically significant.
