# Supplementary figures and images for: Distinct Roles of Extracellular Domains in the Epstein-Barr Virus-Encoded BILF1 Receptor for Signaling and Major Histocompatibility Complex Class I Downregulation
Source: mBio. 2019 Jan 15;10(1):e01707-18. doi: 10.1128/mBio.01707-18 (PMC6336419; doi:10.1128/mBio.01707-18)

A

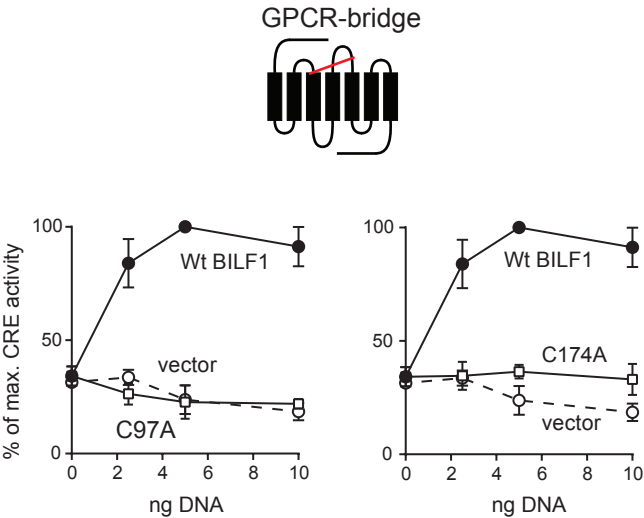

B

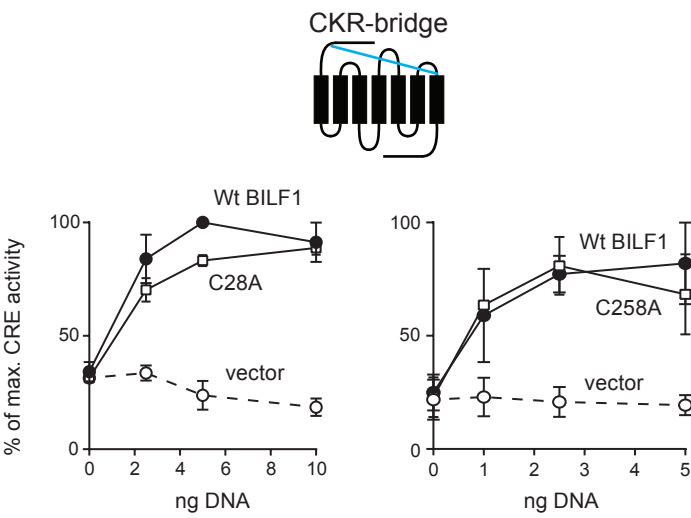

Supplement: FIG S1 [file mBio.01707-18-sf001.pdf]

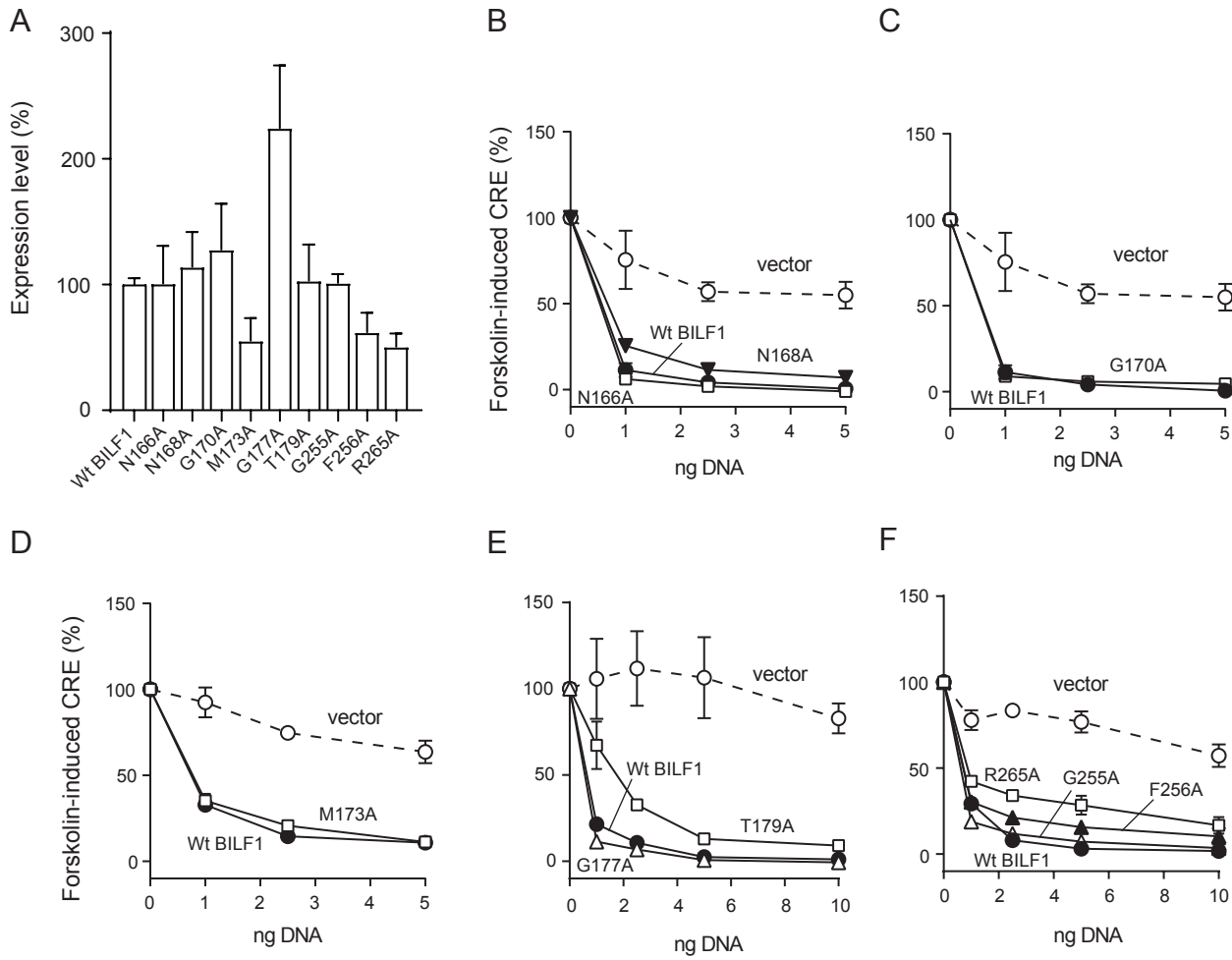

Supplement: FIG S2 [file mBio.01707-18-sf002.pdf]

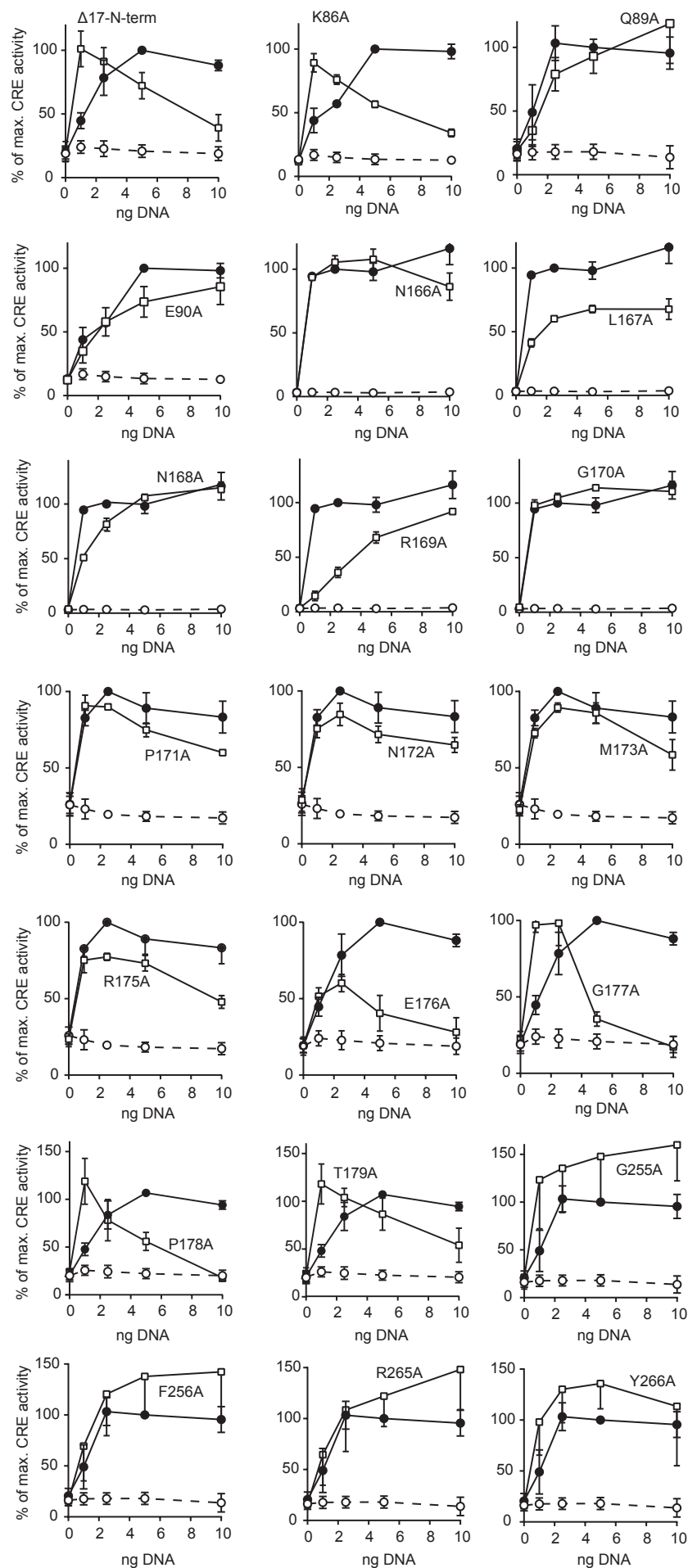

Supplement: FIG S3 [file mBio.01707-18-sf003.pdf]

Supplementary figure 4

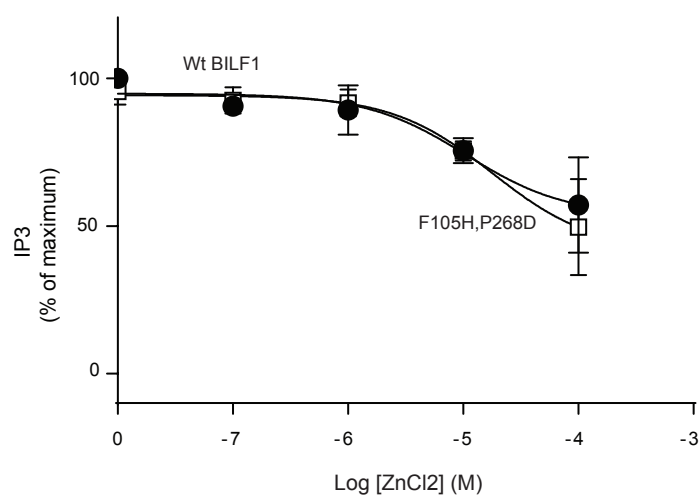

Supplement: FIG S4 [file mBio.01707-18-sf004.pdf]
